# Non-invasive estimation of the powder size distribution from a single speckle image

**DOI:** 10.1038/s41377-024-01563-6

**Published:** 2024-08-21

**Authors:** Qihang Zhang, Ajinkya Pandit, Zhiguang Liu, Zhen Guo, Shashank Muddu, Yi Wei, Deborah Pereg, Neda Nazemifard, Charles Papageorgiou, Yihui Yang, Wenlong Tang, Richard D. Braatz, Allan S. Myerson, George Barbastathis

**Affiliations:** 1 Department of Electrical Engineering and Computer Science, Massachusetts Institute of Technology, Cambridge, MA 02139 USA; 2 Singapore-MIT Alliance for Research and Technology (SMART) Centre, Singapore, 117543 Singapore; 3 Department of Chemical Engineering, Massachusetts Institute of Technology, Cambridge, MA 02139 USA; 4 Department of Mechanical Engineering, Massachusetts Institute of Technology, Cambridge, MA 02139 USA; 5 Process Chemistry Development, Takeda Pharmaceuticals International Co, 40 Landsdowne St, Cambridge, MA 02139 USA; 6 ShinrAI Center for AI/ML, Data Sciences Institutes, Takeda Pharmaceuticals International Co, 650 E Kendall St, Cambridge, MA 02142 USA; 7 Present Address: Department of Precision Instruments, Tsinghua University, Beijing, 100084 China

**Keywords:** Imaging and sensing, Optical metrology

## Abstract

Non-invasive characterization of powders may take one of two approaches: imaging and counting individual particles; or relying on scattered light to estimate the particle size distribution (PSD) of the *ensemble*. The former approach runs into practical difficulties, as the system must conform to the working distance and other restrictions of the imaging optics. The latter approach requires an inverse map from the speckle autocorrelation to the particle sizes. The principle relies on the pupil function determining the basic sidelobe shape, whereas the particle size spread modulates the sidelobe intensity. We recently showed that it is feasible to invert the speckle autocorrelation and obtain the PSD using a neural network, trained efficiently through a physics-informed semi-generative approach. In this work, we eliminate one of the most time-consuming steps of our previous method by engineering the pupil function. By judiciously blocking portions of the pupil, we sacrifice some photons but in return we achieve much enhanced sidelobes and, hence, higher sensitivity to the change of the size distribution. The result is a 60 × reduction in total acquisition and processing time, or 0.25 seconds per frame in our implementation. Almost real-time operation in our system is not only more appealing toward rapid industrial adoption, it also paves the way for quantitative characterization of complex spatial or temporal dynamics in drying, blending, and other chemical and pharmaceutical manufacturing processes.

## Abstract (web summary)

We accelerate a speckle-based particle size detection method by engineering the incident pupil shape. This high-speed probe is capable to detect the particle size evolution in fast dynamical systems.

## Graphical abstract

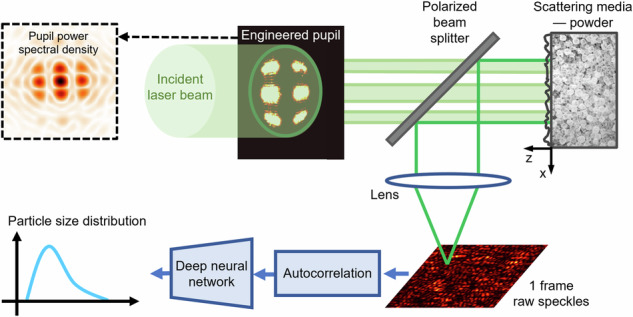

## Introduction

The phenomenon of coherent scattering forming speckle patterns from a rough surface has been recognized since the 1960s. Initial analyses associated the statistical properties of speckle with the roughness statistics of the subwavelength granularities^1,2^. As a known source of artifacts in coherent imaging systems, speckle has been the focus of many studies aimed at suppressing its impact via signal processing in coherent tomography^3–7^, fluorescence microscopy^8,9^ and imaging through diffuse media^10–17^. A well-known speckle correlation property, the “memory effect”, is extensively employed to despeckle by exploiting statistical homogeneity within small neighborhoods of angular directions without necessitating consideration of the physical properties of the scattering material itself^13,14^.

The opposite attitude of exploiting laser speckles to non-invasively characterize surfaces has also been widely pursued^12,18–24^. The prevalent method involves reconstructing the amplitude and phase of the optical field, which is notoriously difficult^2,25,26^ due to the multitude of vortices in the speckle pattern. Conventional closed-form formulas require the condition that the surface height fluctuation be less than or comparable to the light wavelength^1,2^, which is not applicable in many industrial processes. Alternatively, end-to-end machine learning models are capable of qualitatively categorizing various materials based on the appearance of the scattered light^24,27–29^.

Recently, we established a quantitative relationship between the speckle autocorrelation and the granularity of the powder for particle sizes significantly larger than visible wavelengths, ranging from 50 μm to 1 mm. The overall approach estimates the particle size distribution (PSD) from the backscattered light using a Physics-Enhanced Autocorrelation-based Estimator (PEACE)^30^. To our knowledge, the PEACE technique provided the first non-invasive, in-line and quantitative particle size analysis for dense powder surfaces. However, it necessitates multi-frame data collection, and the total measurement time, including data collection and computation, is approximately 15 s. This might be very long for many applications, especially while the powders are being agitated.

In this report, we propose a new PSD estimation method, based on pupil engineering, which overcomes the need for multiple frames. Our learning-based model can estimate the powder size distribution from a single snapshot speckle image, consequently reducing the reconstruction time from 15 s to a mere 0.25 s. Pupil engineering, a common method for shaping the point spread function and the corresponding power spectral density, is typically utilized for super-resolution microscope^31–33^, compressive imaging^34–37^, spectral imaging^38,39^ and 3D localization^40–43^. In our context, pupil engineering is a modulation of the beam profile that is incident on the powder. This enhances the sidelobes of the speckle correlation function, where size information is obtained from, resulting in a more robust measurement.

We analyzed the sensitivity of the PSD for both clear and engineered pupils. Although we drop two-thirds of the photons with an intensity mask, the remaining photons are redirected to a more advantageous spatial distribution, facilitating particle size estimation from a single frame instead of 200 frames in the original PEACE^30^, and yielding improved performance overall. Moreover, after the neural network training process, estimation from a single image requires fewer computational resources it can be processed on a CPU, while the ensemble average of autocorrelations needs a GPU for a practical speed.

Numerous manufacturing and research processes pose a strong demand for swift and non-invasive surface characterization^23,30,44–51^. In particular, the pharmaceutical industry, to ensure product uniformity, requires monitoring of the particle size distribution during the drying process^30,44,45^. To demonstrate the effectiveness of quick size estimation, we conducted a pilot drying experiment. A cost-effective slice of the 3D-printed intensity mask can push the response time of this non-invasive surface characterization technique into the real-time domain. This development broadens its potential applicability beyond pharmaceutical manufacturing to other industries such as batteries^46–48^, rock deterioration^52–55^, construction materials^50,51^, food security^49^ and paper identification, e.g. currency verification^23^.

## Results

### Speckle correlation enhancement by pupil engineering

**Fig. 1:**
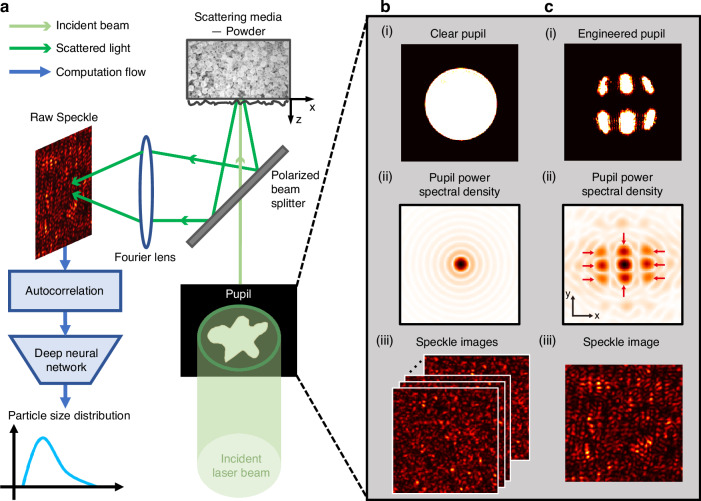
**Laser speckle apparatus with the pupil engineering**. **a** Schematic representation of our speckle granularity probe. An engineered pupil shapes the incident beam into a designed pattern. The powder surface scatters the light in all directions, which is subsequently collected using a lens. For simplicity, we omit a quarter waveplate between the powder surface and the polarized beam splitter in this diagram, detailed information can be found in Supplementary Section 4. The raw images are processed by the autocorrelation function before entering a neural network for PSD estimation. **b** (**i**) The original clear beam profile with a 5 mm diameter. (**ii**) The power spectral density of the clear beam, with the zero-order lobe holding 87.4% of the photon energy. (**iii**) An example of the speckle pattern for the clear pupil, demonstrating statistical symmetry in all directions. **c** (**i**) The engineered pupil on the powder surface, blocking approximately two-thirds of the total photons. The feature size is in millimeter scale which is larger than our interest particle size range. As later mentioned in Discussion, mild diffraction effects are evident by the time the light reaches the powder, but this does not affect our application. (**ii**) Its power spectral density has eight stronger side lobes (marked by red arrows) compared to **b(ii)**, and the peak radial positions along the x and y directions are different. (**iii**) The modified speckle image displays asymmetric spatial frequency

Figure 1a presents the sketch of our laser speckle probe. We positioned a 3D-printed intensity mask in the path of the incident beam to alter its original clear profile into a designed pattern and then gathered the light scattered from the powder surface. The autocorrelation function, once processed, enables a neural network to estimate the particle size distribution. We developed a forward model, expanding upon previous work^30^, which yields the relationship between autocorrelation function $\left\langle A\left({\boldsymbol{u}}\right)\right\rangle$ and PSD $p\left(r\right)$ as

1 $$\left\langle A\left({\boldsymbol{u}}\right)\right\rangle ={\left|M\left({\boldsymbol{u}}\right)\right|}^{2}\frac{{\left|\int {r}^{2}{\rm{Jinc}}\left(r\left|{\boldsymbol{u}}\right|\right)p\left(r\right){{\rm{d}}{r}}\right|}^{2}}{{\left|\int {r}^{2}p\left(r\right){{\rm{d}}{r}}\right|}^{2}}$$

The detailed derivation is in Supplementary Section 1. The normalized displacement ${{{\boldsymbol{u}}}}\,{=}\,{{{\boldsymbol{u}}}}^{{{{\prime}}}}{\boldsymbol{/}}\lambda f$ relates to the spatial displacement ${{\boldsymbol{u}}}^{{{\prime}}}$, $\lambda$ is the wavelength and $f$ is the focal length of the Fourier lens as shown in Fig. 1a. The ensemble average is denoted by $\left\langle \cdot \right\rangle$. The function ${\rm{Jinc}}\left(x\right)=2{J}_{1}(x)/x$, where ${J}_{1}(x)$ is the first order Bessel function of the first kind. The particle size term manifests itself as modulation in the contrast of the autocorrelation sidelobes: larger particle size results in a weaker sidelobe intensity. ${\left|M\left({\boldsymbol{u}}\right)\right|}^{2}$ is the power spectral density for the modulus square pupil function ${{\rm{|}}m\left(x\right){\rm{|}}}^{2}$. This term implies that only an intensity mask can modify the speckle pattern; a phase mask is ineffective, because the phase is rendered useless by additional random modulation by the fine roughness on the particle surface.

Figure 1b(ii) and 1c(ii) illustrate the power spectral density ${\left|M\left({\boldsymbol{u}}\right)\right|}^{2}$ for the original clear pupil Fig. 1b(i) and the engineered pupil Fig. 1c(i), respectively. Figure. 1b(ii) is the well-known Airy pattern in which more than 87.4% of photon energy is concentrated in the non-informative main lobe, while the peak intensity of the useful sidelobe accounts for a mere 0.2% of that of the main lobe. Consequently, for the conventional clear pupil where the sidelobes drop sharply according to an inverse square law, the inverse process becomes severely noise sensitive and requires extensive averaging. The engineered pupil retains only one-third of the photons compared to the original beam, but the main lobe’s contribution in the power spectral density is reduced to 35.05%, resulting in markedly stronger sidelobes, as shown in Fig. 1c(ii). The engineered effective sidelobe energy increases to 62.9% from 9.0% for the clear pupil. Figure. 1b(iii) and Fig. 1c(iii) provide examples of the raw speckle pattern captured from the camera. The texture in Fig. 1c(iii) exhibits more elongated features compared to that of Fig. 1b(iii), indicating that photons are redirected into a designed spatial distribution.

**Fig. 2:**
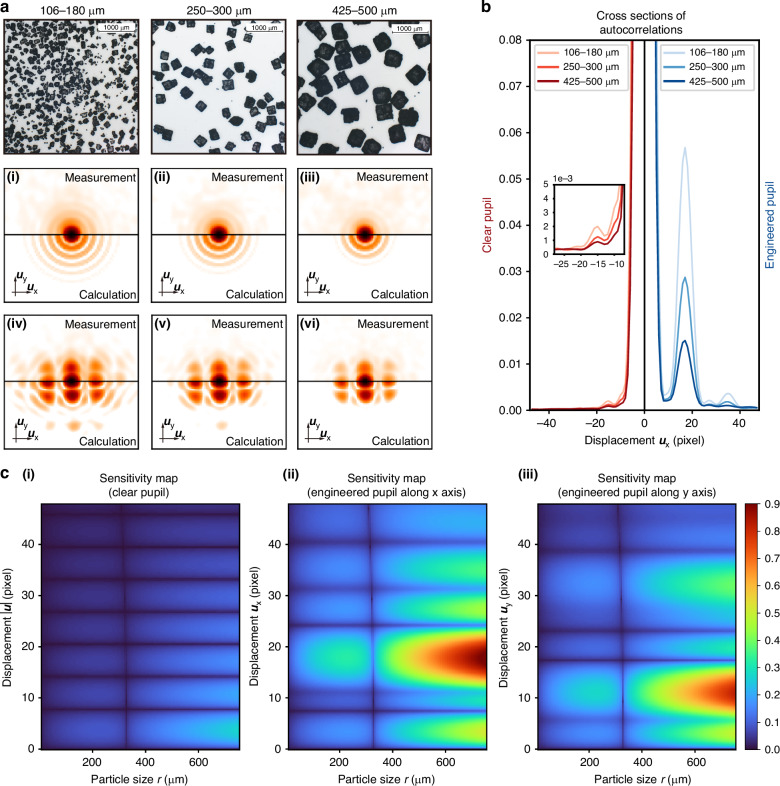
**Microscopic images and the corresponding average autocorrelations for KCl powder of different sizes**. **a** (**i**)–(**iii**) Autocorrelations of the clear pupil for sizes 106–180 μm, 250–300 μm and 425–500 μm. (**iv**)–(**vi**) The same plots for engineered pupils. The upper half depicts the measurements averaged over 500 frames, while the lower half presents calculations from Eq. (1). They show a high degree of consistency, with only minor discrepancies. To enhance the visibility of the sidelobes, the autocorrelation values are raised to the power of one-fourth. **b** Horizontal cross sections along the center (the black line in (**a**) (**i**)–(**vi**)). y-axis is the raw autocorrelation value. The inset is a zoomed-in view of the clear pupil. **c** Sensitivity $S({\boldsymbol{u}},r)$ of the ensemble averaged autocorrelation $\left\langle A\left({\boldsymbol{u}}\right)\right\rangle$ to the population of particles of size *r* for a sample set with the size ranging from 250 μm to 300 μm, according to Eq. (2). (i) Clear pupil, corresponding to (**a**) (**ii**), (**ii**), (**iii**) Engineered pupil along x axis (ii) and y axis (iii), corresponding to **a**(v). For better visibility, ${{|S}({\boldsymbol{u}},r)|}^{1/4}$ has been plotted

Figure 2a presents a comparison of the averaged speckle autocorrelations of varying sizes between the natural clear pupil (i)–(iii) and the engineered pupil (iv)-(vi). The measurement result is depicted in the upper half of each subplot, while the lower half represents the calculation derived from Eq. (1). For both types of pupils, the sidelobe intensity shows a decay as the particle size increases, which conforms to the prediction from our forward model. The 1-st order sidelobe (around pixel -10 labeled in Fig. 2b) merges in the main lobe for the original pupil and becomes indistinguishable, while for the engineered pupil, it is clearly visible in Fig. 2a(iv)–(vi) along the y direction. Figure 2b shows the horizontal cross-sections across the center in Fig. 2a. Both the absolute value and the size modulation contrast increase with the engineered pupil. Specifically, the 2-nd order sidelobe intensity for the 106–180 µm sample increased from 0.002 to 0.055. Higher-order sidelobes are hard to resolve for the clear pupil while some features can still be observed for the engineered one. The overall improvement is ~25–45 times enhancement for different sidelobes.

We defined the measurement sensitivity $S\left({\boldsymbol{u}},r\right)=\partial \left\langle A\left({\boldsymbol{u}}\right)\right\rangle /\partial p\left(r\right)$ to quantify how the autocorrelation at displacement ${\boldsymbol{u}}$ is modified if a small population of particles of size $r$ are added to the powder. The result is

2 $$S\left({\boldsymbol{u}},r\right)=\left\langle A\left({\boldsymbol{u}}\right)\right\rangle \left(\frac{{r}^{2}{\rm{Jinc}}\left(r\left|{\boldsymbol{u}}\right|\right)}{\int {r}^{2}{\rm{Jinc}}\left(r\left|{\boldsymbol{u}}\right|\right)p\left(r\right){{\rm{d}}{r}}}-\frac{{r}^{2}}{\int {r}^{2}p\left(r\right){{\rm{d}}{r}}}\right)$$

More details about this sensitivity expression are in Supplementary Section 1. Figure 2c presents the sensitivity maps for the 250–300 µm sample set. Smaller particle size always suffers a lower sensitivity in all maps, which is consistent with our discussion in PEACE^30^. Compared to the clear pupil in Fig. 2c(i), the sensitivity is enhanced at all particle size $r$ in Fig. 2c(ii), (iii), and the engineered pupil gains a more expansive and continuous feature region along ${\boldsymbol{u}}$. Given the symmetry of all directions for the particles in the illumination region, the size modulation term is dependent solely on the absolute value of $\left|{\boldsymbol{u}}\right|$ rather than ${\boldsymbol{u}}$. However, we deliberately introduce asymmetry to the pupil to disambiguate spatial frequencies along the *x* and *y* directions. This ensures that a dip along the *x* direction is compensated by a corresponding peak along the *y* direction, when we only consider the radial coordinate. This heuristic strategy of pupil design and accurate values for all designed features are concisely outlined in Supplementary Section 7. Other strategies are certainly possible, but beyond the scope of our present work.

### PSD estimation from a snapshot speckle image with a learning-based model

Inverting Eq. (1) with the clear pupil is a highly ill-posed problem due to two challenges. First, in the size modulation term, the probability size *p*(*r*) carries a weight ${{{r}}}^{2}{\rm{Jinc}}\left({{r}}\left|{\boldsymbol{u}}\right|\right)$. This imbalanced weight approaches 0 as $r$ approaches 0, resulting in ill-posedness for small particles^30^. Second, the presence of weak sidelobes results in low contrast for the size modulation term. The engineered pupil does not solve the first problem, but significantly improves the second, leading to less ill-posedness and, subsequently, less need for regularization and training data.

**Fig. 3:**
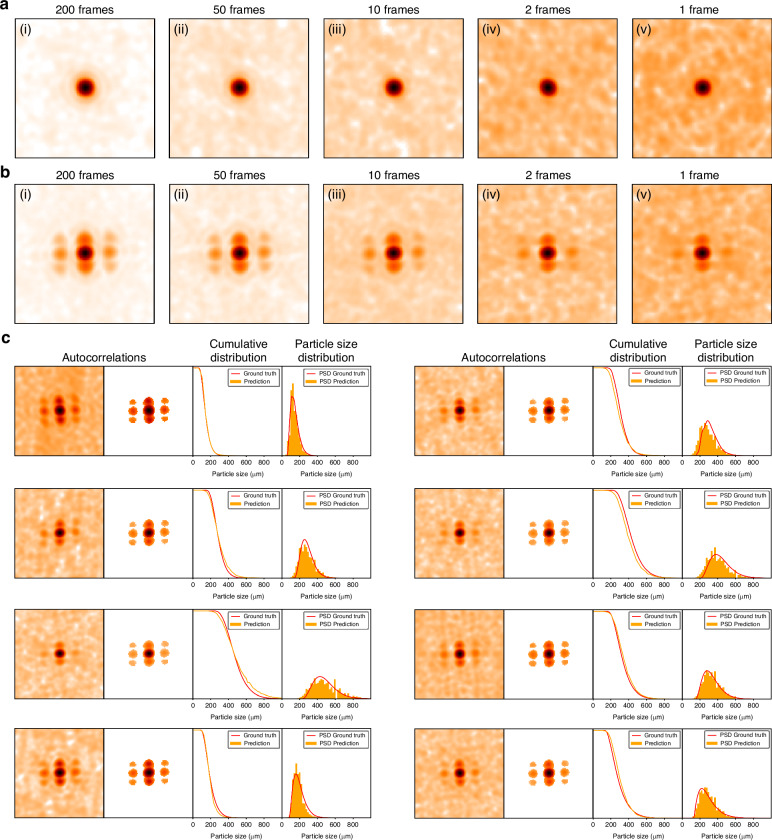
**Single-frame speckle image PSD estimation via machine learning**. Speckle autocorrelations for the KCl sample in the 425–500 μm size range at different averaging frames, with (**a**) the clear pupil and (**b**) the engineered pupil. **c** Test results from the neural network estimation. The first column presents the single-frame autocorrelations, while a digital mask is applied in the second column to address the feature region. The third column is the output cumulative distributions (orange curve) together with their ground truth (red curve). We differentiate it to obtain the estimated PSD, plotted in the last column (orange bar), which aligns well with the corresponding ground truths

**Fig. 4:**
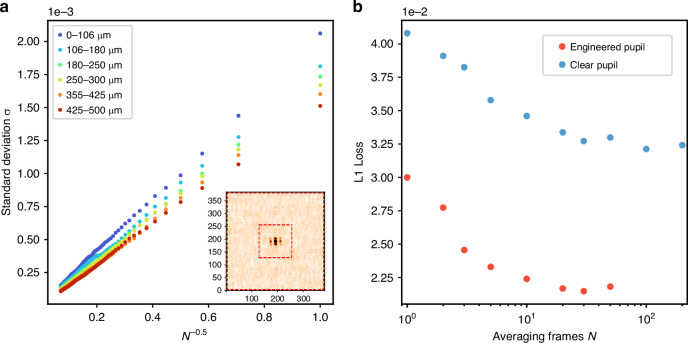
**Analysis of background fluctuations and model performance**. **a** The standard deviation versus the averaging frames $N$. This value is proportional to $1/\sqrt{N}$ and demonstrates stronger fluctuations for smaller particles. The inset shows an autocorrelation example, with two red squares highlighting the region in between used to calculate the standard deviation, thus quantifying the background fluctuation. **b** The optimized test L1 loss for models utilizing an average image from $N$ frames as input. A larger $N$ value improves performance for both pupils, with the engineered pupil consistently outperforming the clear pupil

Figure 3a,b shows the speckle autocorrelations with a reduction in averaging frames from 200 to 1 for the clear pupil and the engineered pupil, respectively. The weak signal in the clear pupil gradually merges into the background fluctuations with fewer frames. For the engineered pupil, some distinguishable features persist above the fluctuations even in a single frame image, which could be utilized to infer the particle size distribution. Figure 4a plots the background fluctuations as a function of averaging frames $N$. We choose the standard deviation in the marked region, as shown in the inset of Fig. 4a, as a quantification of the fluctuation level. It is an incoherent superposition and proportional to $1/\sqrt{N}$. When $N=1$, the fluctuation level is comparable to, or even exceeds, the sidelobes for the clear pupil in Fig. 3a. This analysis teaches us practically how much enhancement is necessary for the sidelobes to remain above the background fluctuations.

As in the original PEACE, a neural network carries out the final step of PSD estimation but now from a single frame autocorrelation. The network takes the autocorrelation as input and its output is the cumulative distribution of the particle sizes. Subsequently, we differentiate this to obtain the PSD. In practice, we discovered that it is helpful to apply a digital filter to select the effective peak region in the input autocorrelations where the peak height is stronger than the background fluctuation. In the normalized pupil power spectral density image, we require that values larger than 0.023 be considered as the effective region. This threshold generates the digital filter. We prepared 18 sample sets of different sizes to form our dataset with each size containing 500 autocorrelation images. The ground truth was calibrated by a commercial offline particle size analyzer, the “Mastersizer”. Half of these sets served as the training dataset, while the rest were employed as test dataset. Additional details about sample preparation and data collection can be found in Supplementary Section 2. The neural network consists of four convolutional stages followed by a fully connected layer and contains around 625k parameters. Initially, the entire network was trained using 9000 synthetic images, generated according to Eq. (1) with different sizes. Subsequently, the parameters in the final three stages were frozen, and only the first stage (containing 4.2k parameters) was fine-tuned using experimental data. This domain transfer technique is widely utilized in training with limited data^56,57^ and offers a better generalization for the size outside the training sets. Additional details about the neural network structure and training hyperparameters are provided in Supplementary Section 3.

Figure 3c presents the test results. All test sets are disjoint in the training process. The first and second columns are the raw and masked autocorrelation images, with the third column depicting the cumulative distribution. We optimize the L1 loss of the cumulative distribution, which is equivalent to the 1-Wasserstein distance, particularly for the 1D probability distribution. The fourth column confirms that the estimated PSD aligns closely with the ground truth PSD across various peak positions and widths. Figure 4b plots the optimized test L1 loss for models that take N frames averaged image as input. Increasing the number of frames results in better predictions. Notably, even when the frame count diminishes to 1, the engineered pupil continues to outperform the 200 frames averaged estimation for the clear pupil.

### Time-lapsed PSD monitoring in a drying process

To demonstrate the capacities of rapid PSD estimation, we implemented a demonstrative drying process^30^ to record the PSD evolution. Detailed specifications of our filter dryer are provided in Supplementary Section 4. The drying process was initiated with 280 g of potassium chloride (KCl) powder and a mixed solvent (40 g of water/60 g of ethanol). Operational conditions were maintained at a temperature of 26 °C, pressure of −720 mbar, and an agitation speed of 4 rpm. The time interval of the PSD measurement reached 0.25 s, including both data collection and computation times. The computer used for this experiment was an Intel Xeon W2245 CPU, 64 GB RAM, we abstained from utilizing the GPU for this measurement, as parallel computation of autocorrelations was not required.

**Fig. 5:**
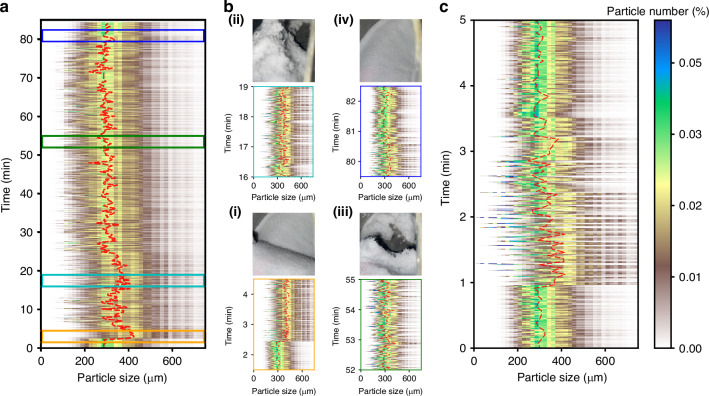
**Monitoring time-lapse PSD in a drying process**. **a** The PSD evolution as a function of time in the potassium chloride (KCl) + mixed solvent (40 g water/60 g Ethanol) drying process. The red dashed line, smoothed for clarity, indicates the PSD peak positions. **b** Zoom-in plots (edge colors are marked in (**a**) and their corresponding camera photos at time intervals of (**i**) 1.5–4.5 min, (**ii**) 16–19 minutes, (**iii**) 52–55 min, and (**iv**) 79.5-82.5 minutes. **c** Time-lapse PSD measurements in the KCl + Acetone drying process. The PSD for the dry powder is shown from 0 to 1 min. The acetone was added into the dryer at 1 min. The PSD returned to its original position after the drying process was completed at 3.5 min

Figure 5a shows the PSD evolution throughout the complete drying process. The red dashed line serves as a smoothed guide to the PSD peak position to enhance visibility. The PSD curve initially shifted right upon the addition of solvents, subsequently reverting to its original positions gradually. Figure 5b provides zoom-in plots and their corresponding camera photos for the regions marked with colorful rectangles in Fig. 5a. Figure 5b(i) captures the starting details, where the PSD remained stable at ~300 μm when initially dry, as depicted in the photo. The addition of solvent at the 2.5-min mark transformed the powder into a slurry. Consequently, the PSD promptly broadened, and the peak shifted to 400 μm. Upon the vacuum’s extraction of the liquid, the slurry transitioned into wet powder, leading to particle agglomeration as large lumps shown in Fig. 5b(ii) camera photo. This phenomenon is referred to as soft agglomeration. The corresponding PSD peak fluctuated between 300 and 400 μm during the 16–19 min interval, with a substantial portion of particles exceeding 450 μm in size. This oscillation is attributed to the drying and subsequent detachment of some powder from the large aggregate. These partially dried, deagglomerated powder particles were smaller in size. The agitator’s continuous movement caused the large aggregate and small dry powder to intermittently intersect with our laser beam spot, resulting in the observed oscillation. The zoom-in plot Fig. 5b(iii) during the 52–55 min interval displays a stronger oscillation centered at 300 μm, due to the progressive deagglomeration of the powder over time. From photo Fig. 5b(iii), we can observe that some fluffy powder fell on the base while other particles remained plump. The zoom-in plot Fig. 5b(iv) during 79.5–82.5 min interval indicates the end of the drying process. At ~81.5 min, the PSD oscillation suddenly stopped, stabilizing at its original position. In Supplementary Section 6, we compared the PSD estimations at the beginning and ending time with the Mastersizer measurements as the ground truth. They match well and both methods validated that the possible crystal breakage^58^ did not happen in this experiment. We did not compare the estimated PSD with the Mastersizer during the drying process due to the Mastersizer’s inability to support in-situ measurement for wet powder, we would either have to wait until it fully dries or add solvent to create a slurry for liquid-mode detection to use Mastersizer. This limitation highlights one of the advantages of our non-invasive measurement over the traditional Mastersizer.

The increased response speed can provide more details for long-duration drying process (tens of minutes). The total duration is influenced by many factors, including the materials, temperature, pressure, and especially the solvents. Typically, organic solvents dry much faster than water and are also commonly used in manufacturing. We demonstrated an additional drying process with Acetone, maintaining all other conditions identical to the previous experiments. The overall drying time is only around 2.5 min, which is too fast to be caught by the old 15-s probe^30^. Figure 5c shows the time-lapse PSD for the Acetone drying experiment. After adding solvent for 1 min, the PSD shifted to the right, reverting to its original position within a minute. By the 3.5-min point, the powder had fully dried. This experiment demonstrated the capability of this technique in the detection of short-duration processes.

## Discussion

Although our pupil design in this work was heuristic, it yielded a significant improvement over PEACE^30^. Due to diffraction and the imperfection in the actual 3D printed mask, the beam intensity profile on the powder surface deforms from the designed shape as shown in Supplementary Section 7. However, this deformation does not influence the main conclusion in this work since the enhancement is enough to distinguish sidelobe peaks from background fluctuations even in a single frame measurement. The use of a neural network is still necessary to compensate for ill-posedness within the integral term in (1), and to further reduce residual noise effects from the enhanced sidelobes, for example due to ambient light or other environmental disturbances. Thus, even further optimization is possible by use of the sensitivity function, but beyond the scope of the present work.

Generalizations for non-spherical particles, as discussed in the clear pupil case^30^, remain applicable here. We also performed a stress test for the bimodal PSD estimation, as outlined in Supplementary Section 5, demonstrating significant improvement over the clear pupil outcome for the same bimodal case^30^. The faster sampling rate allows for quantifying the measured system’s underlying dynamics, e.g. fitting to population balance equations for agglomeration during the drying process^59,60^.

## Materials and methods

The laser model is Excelsior 532 Single Mode with 300 mW output power. The monochromatic camera model is ZWO ASI183MM Pro, which contains 5496 × 3672 pixels with a 2.4 μm pixel size, we run it in the bin-pixel mode with 2744 × 1836 pixels at 45 fps framerate. A 200 μs exposure time was maintained to ensure a high degree of spatial correlation. Commercially obtained potassium chloride (KCl) powder was used to calibrate the speckle. Twenty samples of KCl with varying size distributions were prepared by sieving bulk KCl using sieves attached to the sieve shaker. The sieves, organized in decreasing order of opening size from top to bottom (Sieve opening sizes used: 500 μm, 425 μm, 355 μm, 300 μm, 250 μm, 180 μm, 106 μm). The sieving continued for 15–30 min until the powder weight in each sieves stabilized. Malvern Mastersizer 2000 attached to a Scirocco 2000 dry dispersion unit was used to obtain particle size data. More details about the optics, the filter dryer, the neural network structure, and the material preparation are included in the Supplementary.

The designed masks are fabricated using a commercial 3D printer (Ember 3D printer, Autodesk) with resin (PR48). Layer thickness is set to 50 microns. The printing time is 5 s for each layer, except the first layer which has a longer exposure of 8 s. After printing, the samples are washed with isopropyl alcohol to remove uncured resin from the surface and dried by airflow.

### Supplementary information

Supplementary Information

## Data Availability

All processed data in this study have been deposited in the Harvard Dataverse under accession 10.7910/DVN/21X96Q.

## Code availability

The entire original code has been deposited at https://github.com/qhzhang95/Speckle_pupil-engineering, and is publicly available.
