# Supplementary material for: Non-invasive estimation of the powder size distribution from a single speckle image
Source: Light Sci Appl. 2024 Aug 21;13:200. doi: 10.1038/s41377-024-01563-6 (PMC11339358; doi:10.1038/s41377-024-01563-6)
Supplement: Supplementary file 1 — Supplementary Information [file 41377_2024_1563_MOESM1_ESM.pdf]

Supplementary Information for

Non-invasive Estimation of the Powder Size Distribution from a Single Speckle Image

Authors

Qihang Zhang[1,2,†], Ajinkya Pandit[3], Zhiguang Liu[4], Zhen Guo[1], Shashank Muddu[5], Yi Wei[4], Deborah Pereg[4], Neda Nazemifard[5], Charles Papageorgiou[5], Yihui Yang[5], Wenlong Tang[6], Richard D. Braatz[3], Allan S. Myerson[3], and George Barbastathis[2,4,*]

Affiliations

1. Department of Electrical Engineering and Computer Science, Massachusetts Institute of Technology, Cambridge, Massachusetts 02139, USA.

2. Singapore-MIT Alliance for Research and Technology (SMART) Centre, Singapore 117543, Singapore.

3. Department of Chemical Engineering, Massachusetts Institute of Technology, Cambridge, Massachusetts 02139, USA.

4. Department of Mechanical Engineering, Massachusetts Institute of Technology, Cambridge, Massachusetts 02139, USA.

5. Process Chemistry Development, Takeda Pharmaceuticals International Co, 40 Landsdowne St, Cambridge, Massachusetts 02139, USA.

6. ShinrAI Center for AI/ML, Data Sciences Institutes, Takeda Pharmaceuticals International Co, 650 E Kendall St, Cambridge, Massachusetts 02142, USA.

† Present address: Department of Precision Instruments, Tsinghua University

* Email: gbarb@mit.edu

1. Forward model and background fluctuation analysis

Forward Model Theory.

The forward problem theory establishes a relationship between a specific particle size distribution (PSD) and the raw speckle pattern. It treats the PSD as a probability density function, which determines the ensemble autocorrelation function of the raw speckle. A simplified analytical model is shown in Fig.S1. Without loss of generality, this model assumes that only particles can scatter light and considers the particles as a thin optical mask $a(x)$ with unity reflectivity.

We interpret the particle radius r_i as a random variable distributed according to the PSD $p(r)$. The particle location x_i is also a random variable uniformly distributed across the object plane. $H(x)$ describes the surface height resulting from the randomly placed particles. The corresponding phase of the scattered light is $w(x) = \exp\left(j\frac{2\pi}{\lambda}H(x)\right)$. In the Fraunhofer plane, the electric field $E(x)$ is

$$E(x) = \int e^{j\frac{2\pi}{\lambda f_3}x\xi} S(\xi)d\xi, \tag{S1}$$

where

$$S(\mathrm{x}) = a(x)w(x)\,, \tag{S2}$$

and f_3 is the focal length of the imaging lens. The intensity collected by the CCD camera is

$$I(x) = |E(x)|^2 = \iint e^{j\frac{2\pi}{\lambda f_3}x(\xi_1-\xi_2)} S(\xi_1)S^*(\xi_2)d\xi_1 d\xi_2\,. \tag{S3}$$

We now define the spatial-integral autocorrelation of the speckle image as

$$A(u') = \int I(x)I(x+u')dx\,. \tag{S4}$$

Substituting equations (S2) and (S3) into equation (S4),

$$A(u') = \int \iint \iint e^{j\frac{2\pi}{\lambda f_3}\left[x(\xi_1-\xi_2)+(x+u')(\eta_1-\eta_2)\right]} S(\xi_1)S^*(\xi_2)S(\eta_1)S^*(\eta_2)dx d\xi_1 d\xi_2 d\eta_1 d\eta_2 \tag{S5}$$

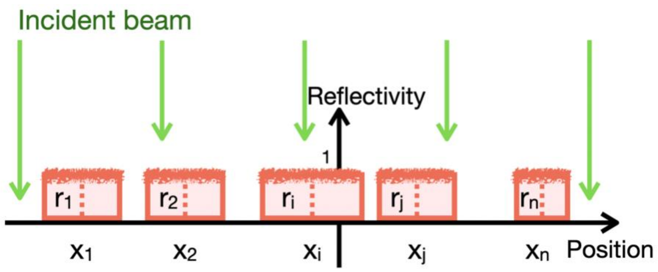

Fig.S1 | A powder model sketch. The sketch only considers the top surface, which is made up of 1D powder particles, represented by red rectangles. Each particle has a radius r_i and a position x_i. The rough top edge indicates that the scattered beam has a random phase at each position on the surface.

After integrating over x,

$$A(u') = \iint \iint e^{j\frac{2\pi}{\lambda f_3}u'(\eta_1-\eta_2)}\, S(\xi_1)S^*(\xi_2)S(\eta_1)S^*(\eta_2)d\xi_1 d\xi_2 d\eta_1 d\eta_2 \int e^{j\frac{2\pi}{\lambda f_3}(\xi_1-\xi_2+\eta_1-\eta_2)x}dx$$

$$= \iint \iint e^{j\frac{2\pi}{\lambda f_3}u'(\eta_1-\eta_2)}\, S(\xi_1)S^*(\xi_2)S(\eta_1)S^*(\eta_2)\,\delta(\xi_1-\xi_2+\eta_1-\eta_2)d\xi_1 d\xi_2 d\eta_1 d\eta_2 \qquad (S6)$$

The delta function sifts the content of the integrand at $\xi_2 = \xi_1 + \eta_1 - \eta_2$. Then the equation can be further simplified as,

$$A(u') = \iint \iint e^{j\frac{2\pi}{\lambda f_3}u'(\eta_1-\eta_2)}\, S(\xi_1)S^*(\xi_1+\eta_1-\eta_2)S(\eta_1)S^*(\eta_2)d\xi_1 d\eta_1 d\eta_2 \qquad (S7)$$

From Equation (S7), we carry out variable substitution as $\eta = \eta_2$, $\sigma = \eta_1 - \eta_2$ and $\tau = \xi_1 - \eta_2$.

$$A(u') = \iint \iint e^{j\frac{2\pi}{\lambda f_3}u'(\sigma+\eta-\eta)}\, S(\eta+\tau)S^*(\sigma+\eta+\tau)S(\eta+\sigma)S^*(\eta)d\tau d\sigma d\eta$$

$$= \int d\tau \left[\int e^{j\frac{2\pi}{\lambda f_3}u'(\sigma+\eta)} S^*(\sigma+\eta+\tau)S(\sigma+\eta)d\sigma\right]\left[\int e^{-j\frac{2\pi}{\lambda f_3}u'\eta} S(\eta+\tau)S^*(\eta)d\eta\right]$$

$$= \int d\tau \left[\int e^{j\frac{2\pi}{\lambda f_3}u'\sigma} S^*(\sigma+\tau)S(\sigma)d\sigma\right]\left[\int e^{-j\frac{2\pi}{\lambda f_3}u'\eta} S(\eta+\tau)S^*(\eta)d\eta\right]$$

$$= \int d\tau \left|\int e^{j\frac{2\pi}{\lambda f_3}u'\sigma} S(\sigma)S^*(\sigma+\tau)d\sigma\right|^2$$

$$(S8)$$

Let $u = \frac{2\pi u'}{\lambda f_3}$,

$$A(u) = \int d\tau \left| \iint e^{ju\sigma} S(\sigma) S^*(\sigma + \tau) d\sigma \right|^2 \quad (S9)$$

After substituting S from equation (S2),

$$A(u) = \int d\tau \left| \iint e^{ju\sigma} a(\sigma) a^*(\sigma + \tau) w(\sigma) w^*(\sigma + \tau) d\sigma \right|^2 \quad (S10)$$

In the internal integral over σ, the phase term $w(\sigma) = \exp\left(j \frac{2\pi}{\lambda} H(\sigma)\right)$ varies much faster than $a(\sigma)$nd $e^{ju\sigma}$, so we can apply the rotating wave approximation to move $w(\sigma)w^*(\sigma + \tau)$ outside the integral over σ.

$$A(u) = \int d\tau \, W(\tau) \left| \iint e^{ju\sigma} a(\sigma) a^*(\sigma + \tau) d\sigma \right|^2, \quad (S11)$$

where $W(\tau) = \langle w(\sigma) w^*(\sigma + \tau) \rangle_\sigma$ is the spatial average of $w(\sigma)w^*(\sigma + \tau)$ over σ. $W(\tau)$ describes spatial correlation of the surface phase. $W(\tau)$ will drop to 0 if τ is larger than the correlation length. For the ideal rough surface, the correlation length is infinitely small and $W(\tau)$ degrades into $\delta(\tau)$.

According to the reference[1], we can safely assume the $W(\tau) = \delta(\tau)$. Thus, the equation (11) is simplified as,

$$A(u) = \left| \iint e^{ju\sigma} |a(\sigma)|^2 d\sigma \right|^2 \quad (S12)$$

Since $a(\sigma)$ is the reflection envelope of the particles, we further assume $a(\sigma)$ is a binary function, $a(\sigma)^2 = a(\sigma)$,

$$A(u) = \left| \iint e^{ju\sigma} a(\sigma) d\sigma \right|^2 \quad (S13)$$

$A(u)$ becomes the power spectral density of $a(\sigma)$. The following sections will discuss different ways to model $a(\sigma)$.

Random impulse process

For the simplest case, we neglect the particle size and shape. $a(\sigma)$ is a set of points at positions x_1, x_2, …, x_K, which are uniformly distributed in the illumination region with a total number of K. We define the probability distribution as the normalized pupil function $m(x) = \frac{Pupil(x)}{\int pupil(x)dx}$, $Pupil(x)$ is the pupil function.

$$a(x) = \frac{1}{K}\sum_{k=1}^{K} \delta(x - x_k) \qquad (S14)$$

$$A(u) = \left|\int e^{jux} a(x)dx\right|^2 = \frac{1}{K^2}\sum_{k=1}^{K}\sum_{q=1}^{K} e^{i2\pi u(x_k - x_q)} \qquad (1S5)$$

We are interested in the ensemble average of $A(u)$.

$$\langle A(u)\rangle = \langle\frac{1}{K^2}\sum_{k=1}^{K}\sum_{q=1}^{K} e^{i2\pi u(x_k - x_q)}\rangle = \frac{1}{K^2}\sum_{k=1}^{K}\sum_{q=1}^{K}\langle e^{i2\pi u(x_k - x_q)}\rangle \qquad (S16)$$

We need to consider two different sets of terms, $k = q$ and $k \neq q$. In the double summation, there exist K separate terms for which $k = q$, each one contributes unity. These terms contribute $\frac{K}{K^2} = \frac{1}{K}$ in $\langle A(u)\rangle$.

In addition, there are $K^2 - K$ terms having $k \neq q$. x_k and x_q are independent to each other, there

$$\langle e^{i2\pi u(x_k - x_q)}\rangle = \int_{-\infty}^{+\infty} m(x_k) e^{i2\pi u x_k} dx_k \int_{-\infty}^{+\infty} m(x_q) e^{i2\pi u x_q} dx_q = |M(u)|^2 \qquad (S17)$$

$M(u)$ is the Fourier transform of the normalized pupil function $m(x)$. Considering these two sets of terms,

$$\langle A(u)\rangle = \frac{K-1}{K}|M(u)|^2 + \frac{1}{K} \qquad (S18)$$

The ensemble averaged autocorrelation is proportional to the power spectral density of the pupil function, with an offset which is inverse proportional to the total number of particles K in the illumination region.

Linear filtered random impulse process

Considering the finite particle size and shape, we assume the particle at x_k has the envelop h.

$$a(x) = \frac{1}{H(0)} \frac{1}{K} \sum_{k=1}^{K} h(x - x_k) = \frac{h(x)}{H(0)} * \frac{1}{K} \sum_{k=1}^{K} \delta(x - x_k) \quad (S19)$$

$$A(u) = \left| \iint e^{jux} a(x) dx \right|^2 = \frac{1}{K^2} \frac{|H(u)|^2}{|H(0)|^2} \sum_{k=1}^{K} \sum_{q=1}^{K} e^{i2\pi u(x_k - x_q)} \quad (S20)$$

$*$ is the convolution operator, $H(u) = \int_{-\infty}^{\infty} h(x) e^{i2\pi ux} dx$ is the Fourier Transform of particle envelop $h(x)$ and $H(0) = \int_{-\infty}^{\infty} h(x) dx$ is the normalization factor to make sure the total region covered by the particles is fixed to unity. Following the same procedure of equation (S15)-(S18),

$$\langle A(u) \rangle = \frac{K-1}{K} \frac{|H(u)|^2}{|H(0)|^2} |M(u)|^2 + \frac{1}{K} \frac{|H(u)|^2}{|H(0)|^2} \quad (S21)$$

If h follows a random distribution, (S19) and (S20) should be reformulated into,

$$a(x) = \frac{1}{K} \sum_{k=1}^{K} h_k(x - x_k) = \frac{1}{\langle H(0) \rangle} \frac{1}{K} \sum_{k=1}^{K} h_k(x) * \delta(x - x_k) \quad (S22)$$

$$A(u) = \left| \iint e^{ju\sigma} a(\sigma) d\sigma \right|^2 = \frac{1}{K^2} \sum_{k=1}^{K} \sum_{q=1}^{K} H_k(u) H_q^*(u) e^{i2\pi u(x_k - x_q)} \quad (S23)$$

Here we ignore the normalization factor for $h(x)$ for simplicity, we will add it back after we get the conclusion. For most cases, the random distribution of $h_k(x)$ is independent to k.

$$\langle A(u) \rangle = \frac{1}{K^2} \sum_{k=1}^{K} \sum_{q=1}^{K} \langle H_k(u) H_q^*(u) \rangle \langle e^{i2\pi u(x_k - x_q)} \rangle \quad (S24)$$

Based on the discussion related to (S16), we need to group terms in (S24) into two sets. There exist K separate terms for which $k = q$, whose contribution is $\langle |H(u)|^2 \rangle$. For the other $K^2 - K$ terms with $k \neq q$, $\langle H_k(u) H_q^*(u) \rangle = \langle H_k(u) \rangle \langle H_q^*(u) \rangle = |\langle H(u) \rangle|^2$ and $\langle e^{i2\pi u(x_k - x_q)} \rangle = |M(u)|^2$.

The final expression of $\langle A(u) \rangle$ is,

$$\langle A(u) \rangle = \frac{1}{C} \left(\frac{K-1}{K} |\langle H(u) \rangle|^2 |M(u)|^2 + \frac{1}{K} \langle |H(u)|^2 \rangle \right) \quad (S25)$$

$$C = |\langle H(0) \rangle|^2 + \frac{1}{K} (\langle |H(0)|^2 \rangle - |\langle H(0) \rangle|^2) = |\langle H(0) \rangle|^2 + \frac{1}{K} \sigma_H^2(0) \approx |\langle H(0) \rangle|^2 \quad (S26)$$

C is the normalization factor. $\sigma_H(0)$ is the standard deviation when $u = 0$. If we ignore the last term in (S25),

$$\langle A(u)\rangle \approx \frac{|\langle H(u)\rangle|^2}{|\langle H(0)\rangle|^2}|M(u)|^2 \tag{S27}$$

Specifically for our powder, in 1D case, we assume that $h_k(x - x_k) = rect(\frac{x-x_k}{r_k})$, $H(u) = r\ sinc(ru)$. r_k is the particle size for k-th particle. r follows the distribution $p(r)$, which is what we want to know.

$$\langle A(u)\rangle = \frac{|\int r\ sinc(ru)p(r)dr|^2}{|\int r\ p(r)dr|^2}|M(u)|^2 \tag{S28}$$

In 2D case,

$$\langle A(\boldsymbol{u})\rangle = |M(u)|^2 \frac{|\int r^2 Jinc(r|\boldsymbol{u}|)p(r)dr\ |^2}{|\int r^2\ p(r)dr|^2} \tag{S29}$$

This expression is the same as equation (1) in the main text.

<u>Sensitivity Analysis</u>

Let us discretize the particle size distribution as

$$p(r) = \sum_{m=0}^{M-1} p_m \delta\left(\frac{r - r_m}{\Delta}\right), \qquad r_m \equiv m\Delta \text{ for } m = 0,1, \dots, M-1. \tag{S30}$$

Here, Δ is the sampling sensitivity of the particle size distribution, M is the measurement complexity (i.e., the number of parameters that we choose to represent the problem) and $(M-1)\Delta r \equiv r_{max}$, the range of sizes for the measurement. We shall refer to the p_m's as the impulse-like parametric description of the particle sizes. It follows that

$$\frac{\partial \overline{r^2}}{\partial p_m} = r_m^2 \tag{S31}$$

$$\frac{\partial \overline{r^2 \mathrm{Jinc}(ru)}}{\partial p_m} = r_m^2 \mathrm{Jinc}(r_m u) \tag{S32}$$

Here, of course,

$$\overline{r^2} = \int r^2 p(r)dr \tag{S33}$$

$$\overline{r^2 \mathrm{Jinc}(ru)} = \int r^2 \mathrm{Jinc}(ru)p(r)dr \tag{S34}$$

With a slight abuse of notation, let us now define,

$$\left.\frac{\partial\langle A\rangle}{\partial p(r)}\right|_{r=r_m} = \frac{\partial\langle A(u)\rangle}{\partial p_m} \qquad (S35)$$

and allow $\Delta \to 0,\ M \to 0$, while r_{max} remains fixed. Then we obtain the equation. (2) in the main text.

$$\frac{\partial\langle A(\boldsymbol{u})\rangle}{\partial p(r)} = \langle A(\boldsymbol{u})\rangle \left(\frac{r^2 \mathrm{Jinc}(ru)}{\overline{\overline{r^2 \mathrm{Jinc}(ru)}}} - \frac{r^2}{\overline{\overline{r^2}}} \right) \qquad (S36)$$

2. Sample preparation and data collection

To calibrate the speckle, we used commercially available potassium chloride (KCl) powder, which has block-shaped crystals as its morphology. We prepared 18 samples of KCl with different size distributions by sieving bulk KCl using a sieve shaker equipped with sieves of opening sizes (500 µm, 425 µm, 355 µm, 300 µm, 250 µm, 180 µm, and 106 µm). We stacked the sieves in the sieve shaker in order of decreasing sieve opening size from top to bottom. We carried out the sieving process for 15-30 minutes until the weight of each sieve with powder remained constant. We added each sieved, dry powder sample individually to a filter-dryer and stirred at 4 rpm to record 500 frames of the speckle pattern. This process generated 18 calibration data sets corresponding to the sieved KCl samples. To evaluate the generalization ability of our neural network model, we choose 9 sample sets to fine-tune the first stage of the neural network, whereas the remaining 9 sets are the test dataset which is disjoint from the whole training process. Both datasets are kept away from the training process for the rest of the neural network.

Offline particle size analysis with Malvern Mastersizer 2000 attached to a Scirocco 2000 dry dispersion unit was used to obtain PSD data as the ground truth for each KCl sample set. Fig. S2 shows the PSD data obtained from Mastersizer corresponding to each sieved sample. The finite sieves and master-sizer measurement limit the number of datasets we can collect, as they are time-consuming and unrecyclable.

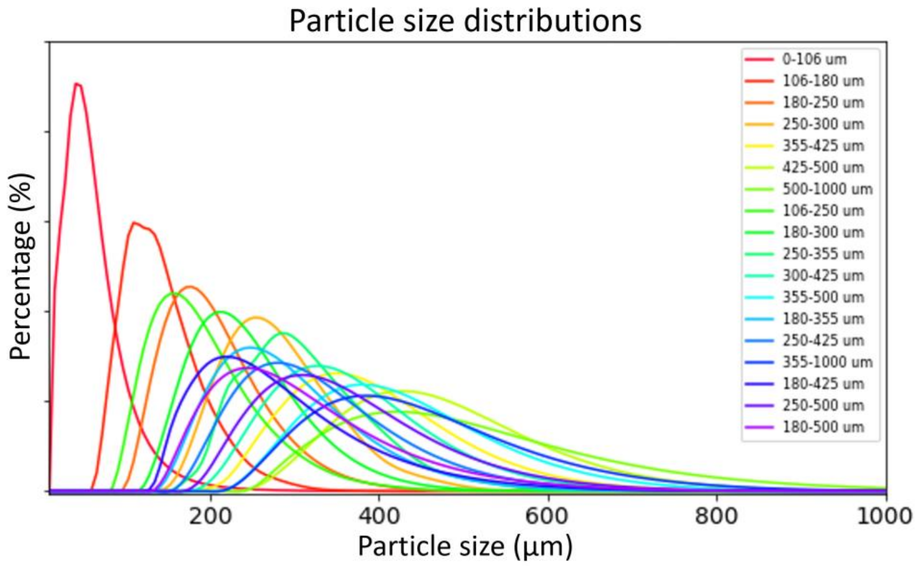

Fig. S2 | PSD calibration curves measured from the Mastersizer serve as the ground truth for each data set.

3. Neural network structure and hyperparameters

The neural network structure is shown in Fig. S3. It consists of four convolutional stages followed by a linear layer. Each stage has four 2D convolutional layers of kernel size 3, combined with Instance-Normalization layer (IN)[2] and ReLU activation. At each stage, the first convolution layer downsamples the image by a factor of 2. The filter depth increases gradually in each stage from 16 to 64. The skip connection structure serves to reduce the gradient vanishing effect[3]. After four convolution stages, the 3D signal is flattened and passed through a linear layer to the output, a non-parametric curve described by 192 samples. This is the cumulative distribution of the particle sizes, which we then differentiate to obtain the PSD. To reduce fluctuations, we bin the pixels of the PSD curve from 192 to 64 pixels.

We pretrain the model (625.5k parameters) with 9000 synthetic autocorrelation-PSD pairs and then fine-tune the first stage (4.2k parameters) with the experimental data. The pretraining and the fine-tuning process have the same hyperparameters. The learning rate was set to be 2×10^{-3} initially and halved whenever validation loss plateaued for 6 consecutive epochs. Batch size was set to 8. The training lasted for 80 epochs with mean absolute error (MAE) as the training loss function. For a 1D probability distribution, MAE of the cumulative distribution is equivalent to the 1-Wasserstein distance of the PSD[4]. The test MAE loss can reach 0.03 for the experimental

test data after fine-tuning. The computer used for training has Intel Xeon G6 CPU, 128 GB RAM, and dual Volta GPUs with 64 GB VRAM. Both training and test data were shuffled before each epoch.

Our calculation consists of three parts: neural network training, autocorrelation calculating and neural network inference. The training process is finished offline, it takes around 6 hours to train with a CPU while a GPU costs 2 minutes. The rest two parts are conducted for the on-line measurement. Since it takes 200 frames data to estimate the PSD with a clear pupil, calculating 200 autocorrelations with CPU takes around 50 seconds in our desktop, while a GPU could compute them in parallel and reduce the computation time down to 0.5 second. With the engineered pupil, it only takes around 0.15 second to calculate single-frame autocorrelation with either a GPU or a CPU. The neural network inference with single frame autocorrelation costs around 0.1 second with a CPU. In summary, GPU is necessary to calculate multi-frame autocorrelations in parallel, which is not required in single-shot PSD estimation.

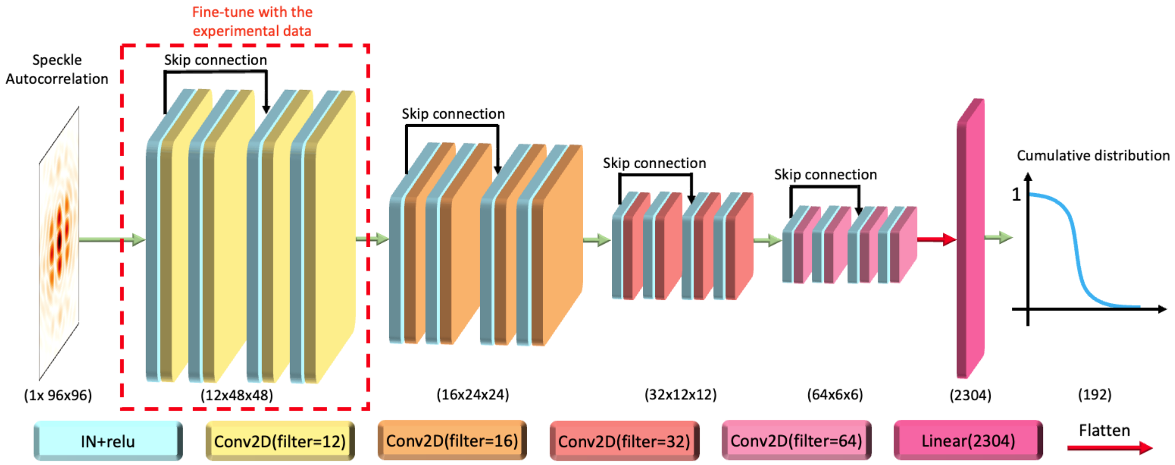

Fig. S3 | Neural Network Structure. The model takes the autocorrelation image as input and the cumulative distributions as output. It consists of four stages with four convolutional layers in each. IN and ReLU activation are applied before each convolutional layer. There is a flattening layer and a linear layer with Sigmoid activation connected to the output of the fourth stage. The output data dimension and the filter parameters for each layer are labeled at the bottom of this figure. We pretrain the model with 9000 synthetic autocorrelation-PSD pairs and then fine-tune the first stage (marked in the red dashed line) with the experimental data. The whole model contains 625.5k parameters while the first stage only has 4.2k parameters.

4. Apparatus and drying experiments

Our apparatus including the optics and the filter dryer is nearly the same as the reference[1], except adding a 3D printed intensity mask on the beam path to shape the pupil.

Optics bench. The wet solid is sealed in the dryer and the laser beam is delivered through a glass window. The angle of incidence on the surface is chosen to be approximately 10 degrees, so as to avoid specular back-reflection from the window onto the camera. The optical beam path is shown in Fig.4(a). The 532 nm laser beam is expanded to 4.8 mm with a beam expander in the telescope configuration, consisting of lenses L1 (focal length 25 mm) and L2 (30 mm). An intensity mask is plugged to shape the beam after L2, with a 10 mm gap. The beam is initially polarized in the direction parallel to the plane shown in the diagram, and so it is reflected by the polarizing beam splitter (PBS) to reach the sample inside the dryer through the glass window. Passage twice through the quarter-wave plate rotates the outgoing polarization direction from parallel to perpendicular so that the scattered light from the powder sample is now transmitted through the PBS and propagates vertically upwards. The wave plate is tilted by approximately 10 degrees for the same reason as the beam, to minimize specular back-reflection. Lens L3 (250 mm) concentrates the scattered light so that an angular range that is as large as possible can be captured by the CCD (model ZWO ASI183MM Pro).

The laser model is Excelsior 532 Single Mode with 300 mW output power. After the fiber coupling, only 230 mW power injects into our system. Our lenses are made of N-BK7 with anti-reflection coating, whose transmission is 99.5% at 532 nm. The beam has a polarization ratio higher than 100:1. The extinction ratio of the polarized beam splitter (PBS) is higher than 1000:1. The transmission of the quarter plate and the silica window are 99.8% and 90%, respectively. The beam power on the sample surface is 203 mW. The power of the scattered light measured at the CCD plane is 120 μW. The coherence length of the laser is 25 m, which corresponds to a temporal bandwidth of 0.01 pm. This results in sufficient temporal coherence to produce sharp speckles.

Image acquisition. The monochromatic CCD model is ZWO ASI183MM Pro with 5496 × 3672 pixels with a 2.4 μm pixel size, we run it in the bin-pixel mode with 2744 × 1836 pixels and 45 fps framerate. 200 μs exposure time is small enough to maintain a high degree of spatial

correlation[1]. The typical size of the speckle pattern calculated from $\lambda f/D$ is 28 µm for our imaging system, which is larger than the pixel size to ensure a good resolution of the speckle pattern.

Filter dryer. The filter drying device shown in Fig.S4(b) was designed based on a prototype[5]. The device has 1400 ml capacity with overall device dimensions of 330 mm height × 220 mm width × 220 mm length without the optical components. The body, impeller, and filter mesh are made of steel and the lid is made of aluminum. The lid contains a vacuum port, an air/gas input port, a camera port, two wash solvent ports, a feed suspension port, and a glass observation window. A vacuum gauge and pressure release valve are attached to the lid. The impeller is connected to a motor on the top of the lid. The impeller blade is designed with teeth to promote cake depumping. An inductive heater and a thermocouple are attached to the bottom of the device to control the drying temperature. This inductive heating plate is used to heat the steel base and the steel body of the dryer. An endoscope camera is attached to the lid to record process videos of the drying material from the top. The optics bench sheds light on the sample and collects the scattered beam through the observation window.

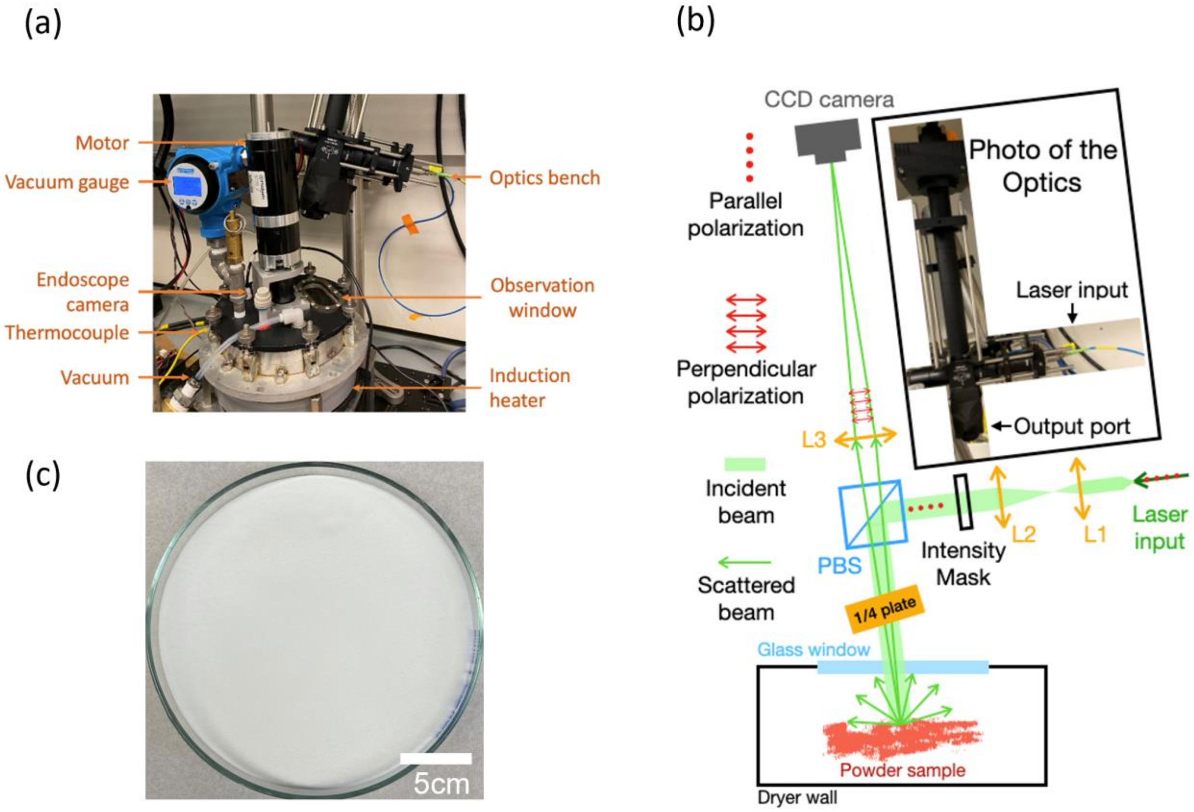

Fig. S4 | (a) The photo of our drying filter. The crucial parts are labeled. **(b)** A sketch of the optics bench. A 3D-printed optical intensity mask is placed on the beam path between Lens L2 and the polarized beam splitter (PBS). Lenses L1, L2, and L3 have focal lengths of 25 mm, 30 mm, and 250 mm, respectively. The distance between the intensity mask and the lens L2 is 10 mm. The inset is the photo of the optics. **(c)** An example of the powder sample.

5. Generality test

The neural network is trained by KCl powder, in principle, dataset collection and network training have to be performed for each new material. However, if the new material has a similar geometry, refractive index, absorption spectrum to KCl, such as sodium chloride (NaCl) powder and, hence, similar statistics for the scattered field, then the neural network trained by KCl should still able to estimate the particle size distribution. We performed a generality test with NaCl powder, maintaining the same sample preparation and measurement procedure. Test results are shown in

Fig.S5, estimated size distributions could fit the ground truth among different test sets, with a slight mismatch for small particles.

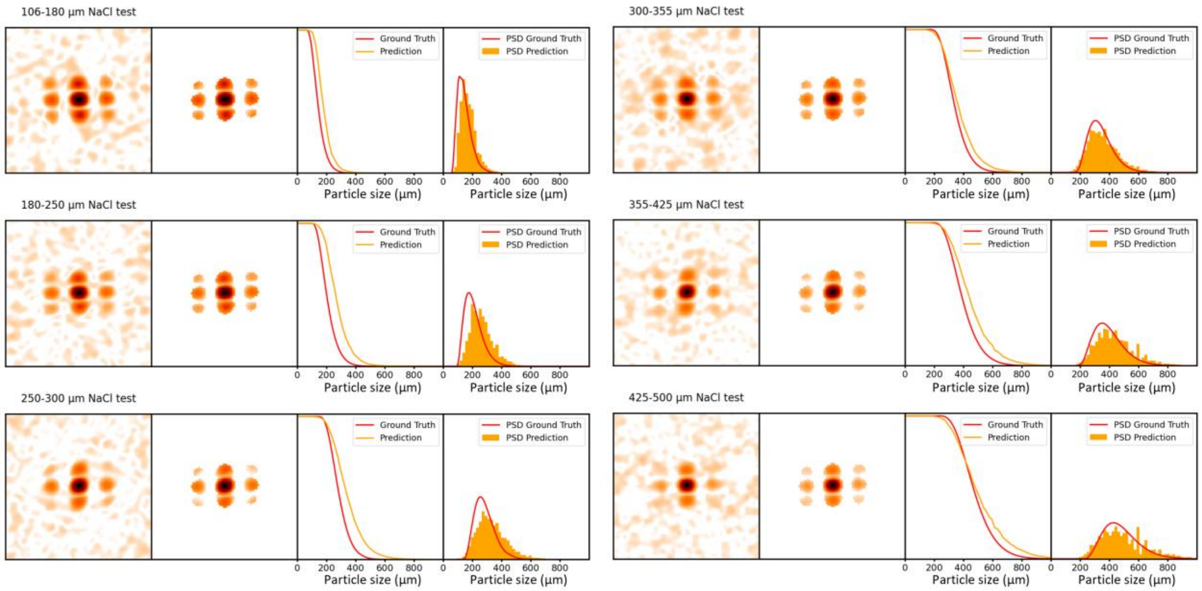

Fig. S5 | Generality test results with NaCl powder. Test results for NaCl power with the model trained by KCl powder. Since two powders have similar physical and chemical properties, the model shows a good generality for NaCl test powder.

We also performed a stress test with bimodal distribution, which completely fails with the clear pupil case discussed in the previous work[1]. There are two reasons: (1) according to equation (1), the bigger particles contribute more to the size modulation term than the smaller ones, so it is harder to catch the smaller peak in the bimodal distribution; (2) Each $|\boldsymbol{u}|$ provides an effective equation to solve the inverse problem, but the power spectral density of the clear pupil is

rotationally symmetric, there are only few individual peaks having enough contrast in the radical coordinate. Therefore, it is too ill-posed to get a bimodal distribution from only few equations.

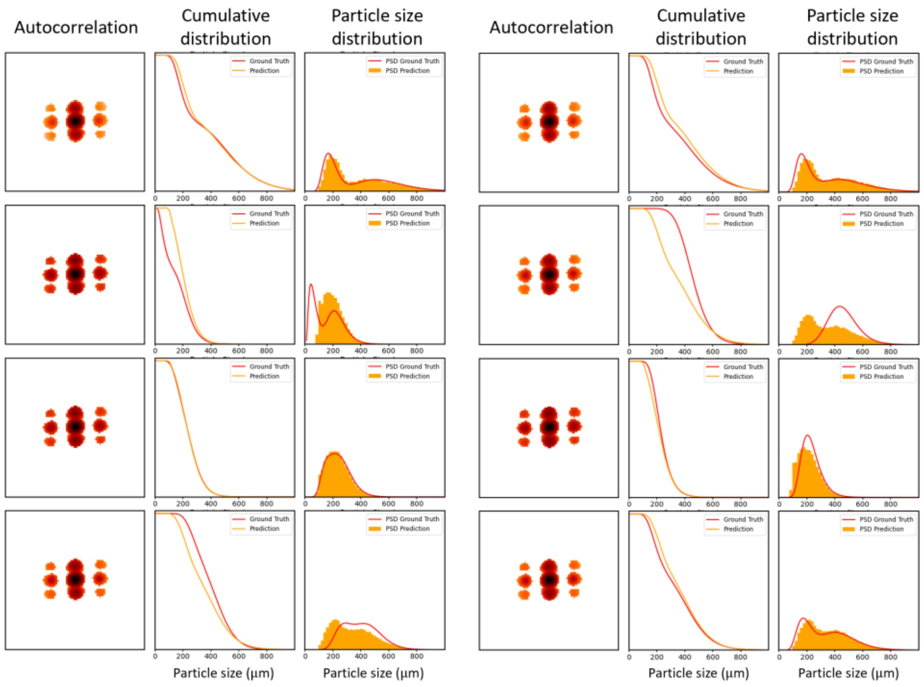

Fig. S6 | Simulation test results including bimodal distributions. The model can fit some of the single peak or bimodal distributions, although there are still some failures. For the failure cases, it always catches the bigger peak and misses the smaller one.

The pupil engineering method helps to release the point (2) because our asymmetric design. It can offer a continuous high-contrast pupil power spectral density in the radical coordinate, resulting in more effective equations to solve the PSD. We trained the neural network with a mixture of simulated single and bimodal autocorrelation-PSD pairs. The test results are plotted in Fig. S6. Although some examples still confuse the model, it is much better than the result in PEACE[1] where the model never figure out the bimodal distributions. For the failure examples, the model can only capture the big peak, which is consistent with the comment (1) in the last paragraph.

6. The estimation and the ground truth of the PSD monitoring

In Fig. S7, we compared the PSD estimations at the beginning and ending time with the Mastersizer measurements as the ground truth. They match well and both methods validated that the possible

crystal breakage[6] did not happen in this experiment. We did not compare the estimated PSD with the Mastersizer during the drying process because Mastersizer does not support *in-situ* measurement for wet powder. We need either wait until it fully dries or add solvent to make a slurry for the liquid-mode detection. It is one of the advantages of our non-invasive measurement compared to the traditional Mastersizer.

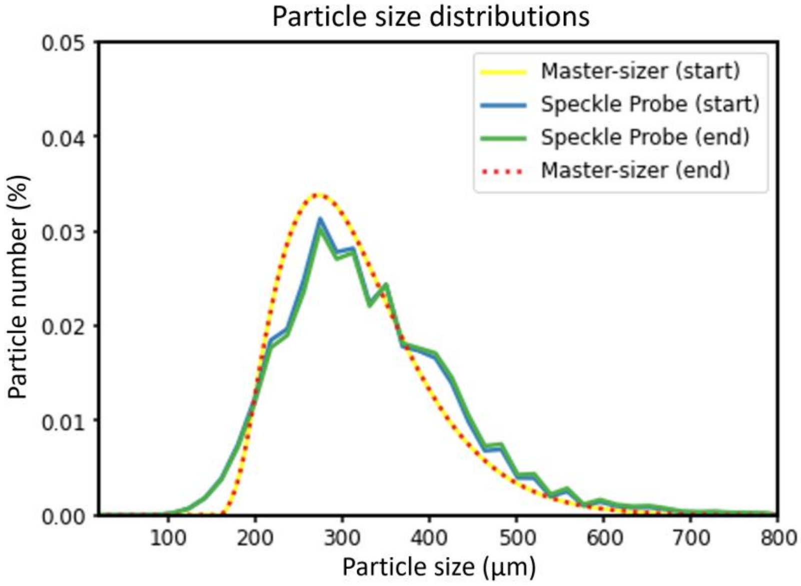

Fig. S7 | Results from the Mastersizer and the speckle Probe. The particle size didn't change after the KCl + EtOH/Water drying process confirmed by both methods and has a peak around 300 µm.

7. Heuristic pupil design strategy

We optimized the pupil in a heuristic way. Fig. S8(a)(i) shows the round-shape pupil with a 4.8 mm diameter. Its power spectral density in (b)(i) is rotationally symmetric and the sidelobe energy is evenly distributed along all directions. Since the size modulation term only depends on the radius coordinate, we can squeeze the energy along the x and y directions to get a stronger sidelobe intensity by a square pupil as shown in (ii). Further introducing a quasi-periodical feature within the illumination region can induce a stronger sidelobe at the corresponding frequency in the power spectral density as shown in (iii). Here we quantitatively optimize the feature period a and the duty

cycle, equivalently, the bar width b. Considering the 1D case, the mask shape in (iii) can be expressed by

$$m(x) = rect\left(\frac{x}{b}\right) + rect\left(\frac{x-a}{b}\right) + rect\left(\frac{x+a}{b}\right)$$

Its Fourier transform is,

$$FT\{m\}(u) = \frac{2}{u} sin\left(\frac{ub}{2}\right)\left(1 + e^{iua} + e^{-iua}\right)$$

$$= \frac{2}{u} sin\left(\frac{ub}{2}\right)(1 + 2\cos{(ua)})$$

Since a is larger than $b/2$, the position of the first sidelobe is determined by $u_1 = 2\pi/a$. The normalized height of the first sidelobe is,

$$s_1 = \frac{|FT\{m\}(u_1)|}{|FT\{m\}(0)|} = \frac{a}{\pi b}\left|sin\left(\frac{\pi b}{a}\right)\right|$$

Since $b < a$, s_1 monotonously decays with b. However, we cannot enhance s_1 by reducing the duty cycle to zero, since we need enough photon energy to activate the CMOS detector, also we would like to cover more area on the sample surface, which is proportional to b, in 1D case. Therefore, we maximize a hand-crafted function L, the product of b and s_1 to balance the sidelobe height and the illumination area.

$$L = bs_1 = \frac{a}{\pi}\left|sin\left(\frac{\pi b}{a}\right)\right|.$$

L reaches its maximum when $b = a/2$.

In Fig.2a(i)-(iii), the first order sidelobe merges into the main lobe due to the motion blur. To avoid merging into the main lobe, the sidelobe position of the engineered pupil is aligned with the middle point of the first and the second sidelobe in the clear pupil with a diameter $D = 4.8$ mm. The position of the first sidelobe for $Jinc(x)$ is $x_1 = 5.3$, the second is $x_2 = 8.5$, so their middle point is $x_m = 6.9 = u_1 D/2$. Thus, the optimized period $a = 2\pi/u_1 = \pi D/(x_m) = 2.18$ mm, and the bar width $b = a/2 = 1.09$ mm, as shown in Fig.S8(a)(iii).

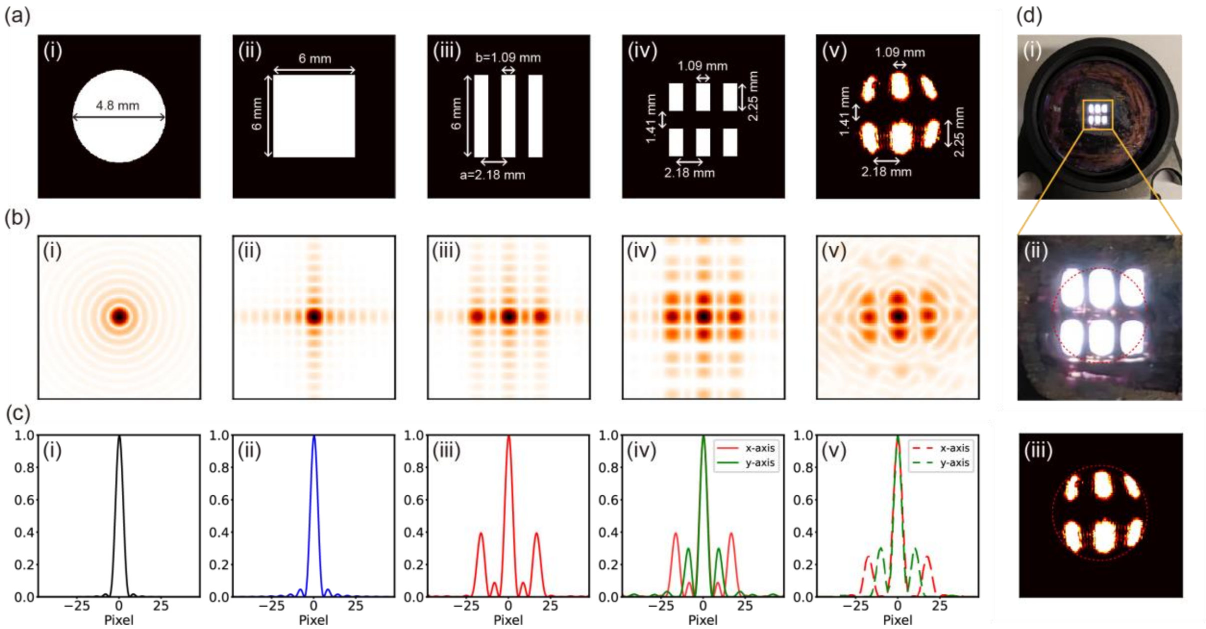

Fig. S8 | Heuristic strategy of the pupil design. The pupil shapes **(a)**, power spectral densities **(b),** and corresponding cross-sections **(c)** at each design step (i)-(v). **(d)** (i) The photo of the 3D printed mask. A lamp is set behind to enhance the visual quality of the pattern. (ii) A zoom-in image of (i). The red circle labels the clear beam shape. Due to the limited printing quality, the fabricated pattern is slightly larger than the clear beam, the region out of the red circle is not illuminated in the experiment. (iii) The illumination pattern at the sample surface. The deformation arises from the aberration in the optical system and the diffraction in the free space.

Moreover, we deliberately make the pupil asymmetric to have different spatial frequencies along the x and y directions as shown in (a)(iv), so that the dip along the x direction is compensated by

the peak along the y direction when we only consider the radial coordinate, it is demonstrated in the power spectral spectrum (b)(iv) and the cross-sections (c)(iv).

We print the designed mask with a commercial 3D printer, and inevitably, this cheap and quick method results in a low quality pattern in the final product shown in (d)(i) and (d)(ii), the pattern at the sample surface is further deformed due to the aberration and diffraction, as presented in (d)(iii). However, this mismatch does not influence the performance much in terms of the sidelobe intensity, as shown in (b)(v) and (c)(v). Quantitively speaking, the strongest sidelobe height in the power spectral density decreases from 0.4 in (c)(vi) to 0.3 in (c)(v), still far larger than 0.01 in (c)(i).

To test the robustness of the designed pupil, we add the Gaussian noise to the pupil pattern with different noise levels σ. Pupil's high-frequency feature has little influence on the low-frequency region of its power spectral density, as shown in Fig.S9, cross-sections of the power spectral density are indistinguishable even for $\sigma = 0.5$. The pupil performance is quite robust to the fabrication quality.

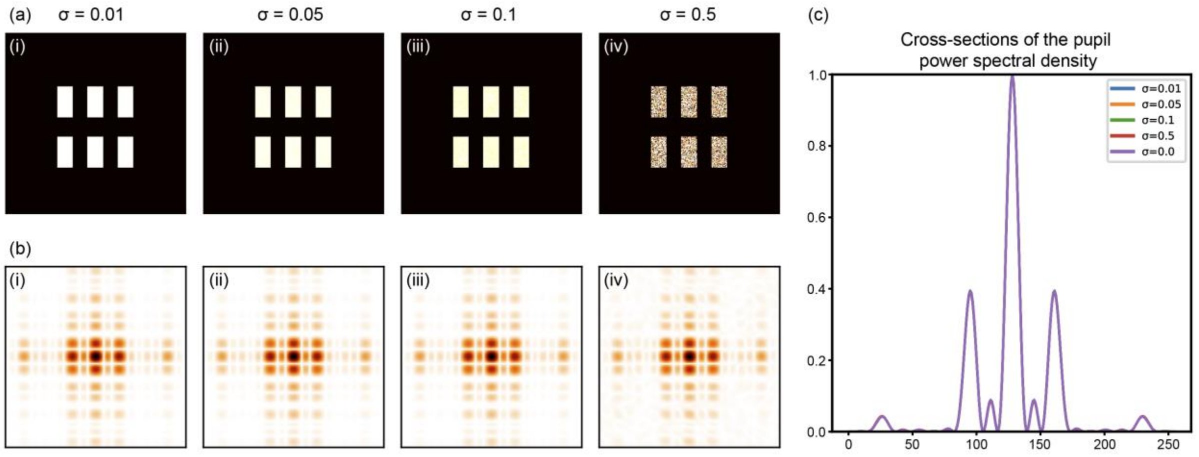

Fig. S9 | Noise test of the designed pupil. (a) The pupil shape with different Gaussian noise level, (i) $\sigma = 0.01$, (ii) $\sigma = 0.05$, (iii) $\sigma = 0.1$, (iv) $\sigma = 0.5$, the original pupil is normalized to 0-1. **(b)** The corresponding power spectral densities. **(c)** Cross-sections of the pupil power spectral density along the horizontal axis. Curves overlap with each other and become indistinguishable.

8. Savitzky-Golay filter for cumulative distributions

In Fig.3c, although the overall trend of the estimated PSD matches the ground truth (GT), there are noticeable discrepancies for certain particle sizes, such as some particle sizes in the third row from the left part and the second row from the right part in Fig.3c. The anomaly discrepancies arise from the differentiation of the zig-zag shape artifacts in predicted cumulative distributions at certain particle sizes, originated from the neural network. In practice, there are two methods to sort out the influence of this anomaly effect. The first is to distinguish the artifact from the real signal by tracking the temporal evolution of the measured size distribution, for example, in Fig.5a and Fig.5c, there is an artifact at around 600 μm and it persists along the whole drying process. Moreover, the evolution of particle size distribution should follow the physical model, e.g. population balance equations. Therefore, it is possible to utilize a dynamical physical model to regularize the PSD predictions, which is out of the scope of this work. The second method is to apply a hand-crafted filter to smooth the output cumulative distribution curve. Here we show results from applying the Savitzky-Golay filter to the cumulative distribution with different window widths in Fig.S10. The discrepancies are almost eliminated with a window width larger

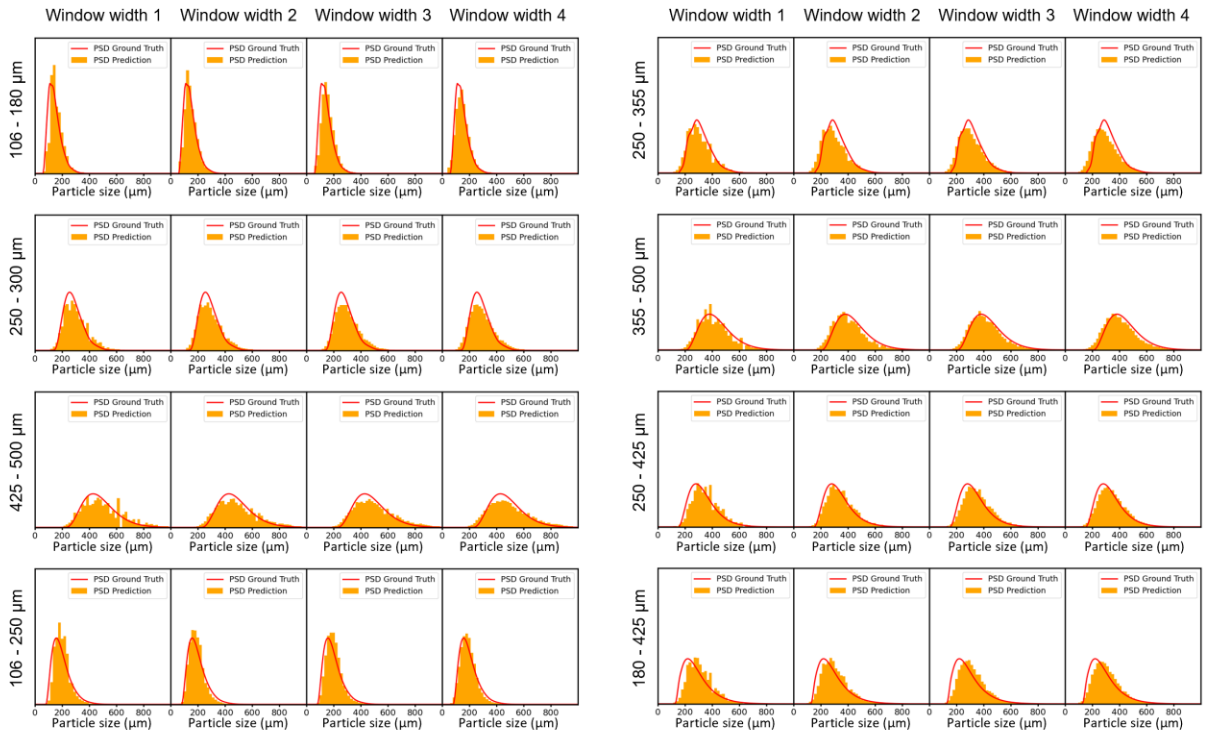

Fig. S10 | Smoothed particle size distributions by the Savitzky-Golay filtering. The output cumulative distributions are smoothed by the Savitzky-Golay filter with different window width, then they are differentiated to get particle size distributions.

than 3. However, the hand-crafted filter doesn't obey any physical prior, so in the main text we have still kept the raw outputs from the neural network and here in the supplement we are showing filtered results as indicative of smoothening options.

Reference

1. Zhang, Q. *et al.* Extracting particle size distribution from laser speckle with a physics-enhanced autocorrelation-based estimator (PEACE). *Nat Commun* **14**, 1159 (2023).
2. Ulyanov, D., Vedaldi, A. & Lempitsky, V. S. Instance Normalization: The Missing Ingredient for Fast Stylization. *ArXiv* **abs/1607.0**, (2016).
3. He, K., Zhang, X., Ren, S. & Sun, J. Deep Residual Learning for Image Recognition. in *2016 IEEE Conference on Computer Vision and Pattern Recognition (CVPR)* 770–778 (2016). doi:10.1109/CVPR.2016.90.
4. Kolouri, S., Pope, P. E., Martin, C. E. & Rohde, G. K. Sliced-wasserstein autoencoder: An embarrassingly simple generative model. *arXiv preprint arXiv:1804.01947* (2018).
5. Capellades, G. *et al.* A Compact Device for the Integrated Filtration, Drying, and Mechanical Processing of Active Pharmaceutical Ingredients. *J Pharm Sci* **109**, 1365–1372 (2020).
6. Lekhal, A. *et al.* Impact of agitated drying on crystal morphology: KCl–water system. *Powder Technol* **132**, 119–130 (2003).
